# Supplementary material for: Ultrastructural diversity and subcellular organization of nigral Lewy pathology in Parkinson’s disease
Source: Nat Commun. 2026 Jun 8;17:7254. doi: 10.1038/s41467-026-74083-z (PMC13396239; doi:10.1038/s41467-026-74083-z)
Supplement: Supplementary file 2 — Reporting Summary [file 41467_2026_74083_MOESM2_ESM.pdf]

nature portfolio

Corresponding author(s): Amanda Lewis

Last updated by author(s): May 26, 2026

Reporting Summary

Nature Portfolio wishes to improve the reproducibility of the work that we publish. This form provides structure for consistency and transparency in reporting. For further information on Nature Portfolio policies, see our Editorial Policies and the Editorial Policy Checklist.

Statistics

For all statistical analyses, confirm that the following items are present in the figure legend, table legend, main text, or Methods section.

n/a	Confirmed	
☐	☒	The exact sample size (*n*) for each experimental group/condition, given as a discrete number and unit of measurement
☐	☒	A statement on whether measurements were taken from distinct samples or whether the same sample was measured repeatedly
☒	☐	The statistical test(s) used AND whether they are one- or two-sided<br>*Only common tests should be described solely by name; describe more complex techniques in the Methods section.*
☒	☐	A description of all covariates tested
☒	☐	A description of any assumptions or corrections, such as tests of normality and adjustment for multiple comparisons
☒	☐	A full description of the statistical parameters including central tendency (e.g. means) or other basic estimates (e.g. regression coefficient) AND variation (e.g. standard deviation) or associated estimates of uncertainty (e.g. confidence intervals)
☒	☐	For null hypothesis testing, the test statistic (e.g. *F*, *t*, *r*) with confidence intervals, effect sizes, degrees of freedom and *P* value noted<br>*Give P values as exact values whenever suitable.*
☒	☐	For Bayesian analysis, information on the choice of priors and Markov chain Monte Carlo settings
☒	☐	For hierarchical and complex designs, identification of the appropriate level for tests and full reporting of outcomes
☒	☐	Estimates of effect sizes (e.g. Cohen's *d*, Pearson's *r*), indicating how they were calculated

Our web collection on statistics for biologists contains articles on many of the points above.

Software and code

Policy information about availability of computer code

Data collection	Leica Thunder Tissue Imager (Leica Microsystems), Tecnai G2 Spirit TEM (ThermoFisher). CM100 Biotwin (Philips), Tecnai Spirit Biotwin (ThermoFisher), 2100 Plus (Jeol), Talos F200C (ThermoFisher).
Data analysis	Leica Application Suite X (RRID:SCR_013673), FIJI (RRID:SCR_002285), IMOD, Amira version 2021.2 (ThermoFisher), EMAN2 (RRID:SCR_016867), Chimera (UCSF).

For manuscripts utilizing custom algorithms or software that are central to the research but not yet described in published literature, software must be made available to editors and reviewers. We strongly encourage code deposition in a community repository (e.g. GitHub). See the Nature Portfolio guidelines for submitting code & software for further information.

Data

Policy information about availability of data

All manuscripts must include a data availability statement. This statement should provide the following information, where applicable:

- Accession codes, unique identifiers, or web links for publicly available datasets
- A description of any restrictions on data availability
- For clinical datasets or third party data, please ensure that the statement adheres to our policy

The raw EM micrographs from the full dataset generated in this study have been deposited in the BioImageArchive (http://www.ebi.ac.uk/bioimage-archive) under accession number S-BIAD3276. Additional data is available upon request.

Research involving human participants, their data, or biological material

Policy information about studies with human participants or human data. See also policy information about sex, gender (identity/presentation), and sexual orientation and race, ethnicity and racism.

Reporting on sex and gender	The biological sex of the post-mortem brain donors was reported in the manuscript, in accordance with the SAGER guidelines.
Reporting on race, ethnicity, or other socially relevant groupings	N/A
Population characteristics	Demographic features and clinical symptoms were abstracted from the clinical files, including sex, age at symptom onset, age at death, disease duration, presence of dementia, core and supportive clinical features for PD and are provided in Supplementary Table 2.
Recruitment	PD brain donors participated in the brain donation program from the Netherlands Brain Bank (www.brainbank.nl). All donors provided written informed consent for a brain autopsy and the use of the material and clinical information for research purposes. Detailed neuropathological and clinical information was made available, in compliance with local ethical and legal guidelines, and all protocols were approved by VU University Medical Center institutional ethics review board (reference number 2019/148). Participants were selceted based on their clinical disease diagnosis and a post-mortem delay of < 7 hours.
Ethics oversight	VU University Medical Center institutional ethics review board (reference number 2019/148). Cantonal Ethics Committee of Vaud (CER-VD; approval numbers 2020-01692 and 2025-01061).

Note that full information on the approval of the study protocol must also be provided in the manuscript.

Field-specific reporting

Please select the one below that is the best fit for your research. If you are not sure, read the appropriate sections before making your selection.

- [x] Life sciences
- [ ] Behavioural & social sciences
- [ ] Ecological, evolutionary & environmental sciences

For a reference copy of the document with all sections, see nature.com/documents/nr-reporting-summary-flat.pdf

Life sciences study design

All studies must disclose on these points even when the disclosure is negative.

Sample size	The sample size of the cohort used for this study was based on the availability of brain donors who participated in the brain donation program from the Netherlands Brain bank between 2017-2022, and presented with a clinical diagnosis of PD and a post-mortem delay of < 7 hours.
Data exclusions	No data were excluded
Replication	Experimental findings were based on replicate observations within multiple patients.
Randomization	No experiments were conducted where randomization was required.
Blinding	Blinding was not relevant to the study

Reporting for specific materials, systems and methods

We require information from authors about some types of materials, experimental systems and methods used in many studies. Here, indicate whether each material, system or method listed is relevant to your study. If you are not sure if a list item applies to your research, read the appropriate section before selecting a response.

Materials & experimental systems

n/a	Involved in the study	
[ ]	[x]	Antibodies
[x]	[ ]	Eukaryotic cell lines
[x]	[ ]	Palaeontology and archaeology
[x]	[ ]	Animals and other organisms
[x]	[ ]	Clinical data
[x]	[ ]	Dual use research of concern
[x]	[ ]	Plants

Methods

n/a	Involved in the study	
[x]	[ ]	ChIP-seq
[x]	[ ]	Flow cytometry
[x]	[ ]	MRI-based neuroimaging

Antibodies

Antibodies used	LB509 Life Technologies # 180215; Clone 42 BD Biosciences #15895639; EP1536Y Abcam # 51253; 11a5 Prothena
Validation	Antibodies were validated by the manufacturers. No additional validation was performed.

Plants

Seed stocks	*Report on the source of all seed stocks or other plant material used. If applicable, state the seed stock centre and catalogue number. If plant specimens were collected from the field, describe the collection location, date and sampling procedures.*
Novel plant genotypes	*Describe the methods by which all novel plant genotypes were produced. This includes those generated by transgenic approaches, gene editing, chemical/radiation-based mutagenesis and hybridization. For transgenic lines, describe the transformation method, the number of independent lines analyzed and the generation upon which experiments were performed. For gene-edited lines, describe the editor used, the endogenous sequence targeted for editing, the targeting guide RNA sequence (if applicable) and how the editor was applied.*
Authentication	*Describe any authentication procedures for each seed stock used or novel genotype generated. Describe any experiments used to assess the effect of a mutation and, where applicable, how potential secondary effects (e.g. second site T-DNA insertions, mosiacism, off-target gene editing) were examined.*
