# Supplementary material for: Ultrastructural diversity and subcellular organization of nigral Lewy pathology in Parkinson’s disease
Source: Nat Commun. 2026 Jun 8;17:7254. doi: 10.1038/s41467-026-74083-z (PMC13396239; doi:10.1038/s41467-026-74083-z)
Supplement: Supplementary file 3 — Transparent Peer Review file [file 41467_2026_74083_MOESM3_ESM.pdf]

nature portfolio

Peer Review File

Ultrastructural diversity and subcellular organization of nigral Lewy pathology in Parkinson’s disease.

Corresponding Author: Dr Amanda Lewis

This manuscript has been previously reviewed at another journal. This document only contains information relating to versions considered at Nature Communications.

This file contains all reviewer reports in order by version, followed by all author rebuttals in order by version.

Version 0:

Reviewer comments:

Reviewer #1

(Remarks to the Author)

The authors have largely addressed my original concerns, and the revision is substantially improved.
At this point I have only two brief, remaining suggestions:
1. Please add one explicit sentence in the Discussion making clear that the proposed “staging” or sequence of Lewy body formation is a plausible model inferred from cross sectional, late-stage material, and not a demonstrated temporal pathway.
2. In the Abstract, please add a short statement that the inclusions were targeted by design using CLEM and that the study is not intended to provide quantitative prevalence or Braak stage–wise comparisons.

Reviewer #2

(Remarks to the Author)
I appreciate the authors' willingness to revise this manuscript. The edits make this revision much improved in terms of how the findings are presented and interpreted. I have just a few minor considerations.

1. For the DAPI signal, the text says that "an intact neuronal nucleus could be clearly identified" but I am not sure I see a nucleus. Is this a typographical error where it should say a nucleus could NOT be clearly identified?

2. In the limitations section of the discussion, it mentions that a-syn oligomers may be below the limit of detection. I suggest the authors also include the fact that post-mortem tissue may under-represent transient meta-stable species where assemblies may dissolve due to post-mortem delay.

Open Access This Peer Review File is licensed under a Creative Commons Attribution 4.0 International License, which permits use, sharing, adaptation, distribution and reproduction in any medium or format, as long as you give appropriate credit to the original author(s) and the source, provide a link to the Creative Commons license, and indicate if changes were made.
In cases where reviewers are anonymous, credit should be given to 'Anonymous Referee' and the source.
The images or other third party material in this Peer Review File are included in the article’s Creative Commons license, unless indicated otherwise in a credit line to the material. If material is not included in the article’s Creative Commons license and your intended use is not permitted by statutory regulation or exceeds the permitted use, you will need to obtain permission directly from the copyright holder.
To view a copy of this license, visit https://creativecommons.org/licenses/by/4.0/

REVIEWERS' COMMENTS

Reviewer #1 (Remarks to the Author):

The authors have largely addressed my original concerns, and the revision is substantially improved.
At this point I have only two brief, remaining suggestions:
1. Please add one explicit sentence in the Discussion making clear that the proposed “staging” or sequence of Lewy body formation is a plausible model inferred from cross sectional, late-stage material, and not a demonstrated temporal pathway.

> ***Response -*** *We have included this statement in the limitations section of our Discussion, which now reads in full:*
>
> Our proposed sequence represents a model inferred from cross-sectional observations of late-stage post-mortem tissue and does not constitute a directly observed temporal progression. It is also important to note that the proposed transmittable species of αSyn, being oligomers or protofibrils (reviewed in[56]), are below the resolution limits of our methods. Additionally, the end-stage nature of our cohort may lead to an underrepresentation of short-lived or early pathological states, particularly given that post-mortem delay and tissue processing may lead to the loss or dissolution of transient, metastable αSyn assemblies. Consequently, static post-mortem observations cannot establish whether distinct ultrastructural phenotypes arise sequentially or independently.

2. In the Abstract, please add a short statement that the inclusions were targeted by design using CLEM and that the study is not intended to provide quantitative prevalence or Braak stage–wise comparisons.

> ***Response -*** *We have included the following sentence in the abstract:*
>
> Because inclusions were selectively targeted, this study focused on mechanistic insight rather than quantitative or disease-stage comparisons.

Reviewer #2 (Remarks to the Author):

I appreciate the authors' willingness to revise this manuscript. The edits make this revision much improved in terms of how the findings are presented and interpreted. I have just a few minor considerations.

1. For the DAPI signal, the text says that "an intact neuronal nucleus could be clearly identified" but I am not sure I see a nucleus. Is this a typographical error where it should say a nucleus could NOT be clearly identified?

> ***Response -*** *It was not a typographical error, however it is true that the nucleus is not obvious in those figures. We have removed this statement to also align with the journal main text guidance to remove all instances of unpublished/not shown data.*

2. In the limitations section of the discussion, it mentions that a-syn oligomers may be below the limit of detection. I suggest the authors also include the fact that post-mortem tissue may under-represent transient meta-stable species where assemblies may dissolve due to post-mortem delay.

> ***Response -*** *We have included a statement to this affect and this section now reads:*
>
> Our proposed sequence represents a model inferred from cross-sectional observations of late-stage post-mortem tissue and does not constitute a directly observed temporal progression. It is also important to note that the proposed transmittable species of αSyn, being oligomers or protofibrils (reviewed in[56]), are below the resolution limits of our methods. Additionally, the end-stage nature of our cohort may lead to an underrepresentation of short-lived or early pathological states, particularly given that post-mortem delay and tissue processing may lead to the loss or dissolution of transient, metastable αSyn assemblies. Consequently, static post-mortem observations cannot establish whether distinct ultrastructural phenotypes arise sequentially or independently.
